# Neonatal seizures in Uyo: the burden, etiological factors and outcome

**DOI:** 10.1186/s12887-025-05742-1

**Published:** 2025-06-06

**Authors:** Eno Etim Nyong, Mkpouto Udeme Akpan, Kingsley Irelosen Akhimienho, Ifunanya Ularinma Ebiekpi, Utibe David David

**Affiliations:** University of Uyo Teaching, Paediatrics, Uyo, Nigeria

**Keywords:** Neonatal, Seizures, Neurological, Term, Subtle

## Abstract

The significant neurological diseases in the newborn can manifest as seizures. Our aim is to establish the burden of neonatal seizures in Uyo, its etiological factors and outcome. This was a retrospective, descriptive, cross-sectional study that was carried out between May 2013 and June 2018. The study was carried out in the neonatal unit of the University of Teaching Hospital, Uyo. Neonates with seizures, suspected seizures or with conditions associated with seizures were identified in the admission register. Information extracted included age at admission, age on onset, gestational ages of neonates, birth weight, mode of delivery, APGAR score, family history of seizures, social status of the parent, significant findings on physical examination. Prevalence rate was taken as the number of neonates with seizures over the total number of neonates admitted during the period. The prevalence of neonatal seizures in Uyo was 2.93%. Majority of the newborns with seizures were term babies. Birth asphyxia and infective causes either singly or in concert with other etiological factors were the important causes of neonatal seizures. Hypocalcaemia as a cause of neonatal seizures tended to show bimodal chronologic distribution. Bizarre movements and other types of subtle seizures were the most common seizure type in the general population. More complications were observed in babies whose periods of onset were within the first three days of life.

**Supplementary Information:**

The online version contains supplementary material available at 10.1186/s12887-025-05742-1.

## Introduction

Significant neurological diseases in the newborn usually manifest as seizures; [1] these seizures can be predictors of adverse neurological outcome [2]. In the general consideration, a seizure is defined as an excessive synchronous discharge originating from the brain, resulting in muscular activity, but this does not strictly apply to the newborns. In this group, seizures are considered as paroxysmal alteration of neurological function which may result in behavioral, motor or autonomic function [3]. The severity of the seizures are mainly dependent on the extent of cerebral insult, the level of maturity of the neonate and the underlying etiology of the seizure. They are frequently occurring neurological problem in the nursery which requires rapid evaluation and treatment to minimize the possibility of associated cardio-respiratory instability and detrimental long term effects.

The epidemiology of neonatal seizures reportedly varies widely [2, 4, 5, 6, 7, 8] and it is not well defined, as it depends on whether it is arrived at using clinic-based study or population-based study. Many studies have documented wide range of prevalence/incidences. The wide range of incidence of neonatal seizures is attributed to the fact that some seizures are indentified through clinical details only, while some are identified with clinical details and Electro-encephalography (EEG) correlates or through EEG only. Some may have clinical episodes with no EEG correlate while some may have been missed because activities that may be mistaken for seizures and many non-epileptic episodes may be misdiagnosed especially as most studies are retrospective studies. The clinical approach does not permit identification of electrical seizures. Clinical-based studies are pre-disposed to reporting higher incidences because the cohort of infants seen are affected by central nervous system (CNS) injuries that may predispose them to seizures [2, 3, 4, 9]. Lower incidences have been reported in some population-based studies [8, 10].

Neonatal seizures are more reported for preterm babies [4, 11, 12, 13, 14] and for low birth weight (LBW) [8, 11, 14]. In most of these instances, the incidence of neonatal seizures have been shown to increase with decreasing gestational age and decreasing birth weight [11, 15]. According to Panayiolopoulous [16], the incidence of seizures in preterm infants is very high and may be as high 57–132/1000 live births. Allan Hill [17] however reported that the incidence of neonatal seizures vary widely and may vary from 0.5% in term babies to 20% in preterm babies based on clinical evidence. These figures were found to increase to 40% when long-term EEG recordings were done in pharmacologically paralyzed term newborns.

Neonatal seizures are one of the morbidities in the newborn period and if not properly managed may lead to mortality. The incidences and prevalence of neonatal seizure that have been reported in some part of the South-South zone of Nigeria are 3.15 per 1000 live birth by Omene [18] in Benin and prevalence of 4.1 was reported in Calabar in 1995 by Asindi et al. [19] and 5% in a revision study eleven years later by Udo et al. [20] in the same study center. Kuti et al. [7] documented a prevalence of 16.2% in the South-West. The size of the problem is not known in Uyo and the true estimate in Nigeria is not known. The study therefore sought to establish the burden of neonatal seizures in Uyo, its etiological factors and outcome.

## Methodology

### Design

This was a retrospective, descriptive, cross-sectional study carried out over a period of May 2013 to June 2018.

### Study location

The study was conducted in the neonatal unit (SPECIAL CARE BABY UNIT SCBU - inborn and SICK BABY UNIT, SBU - outborn) of the University of Uyo Teaching Hospital (UUTH), Uyo. UUTH is located about 6 km from the centre of the town. The population of Akwa Ibom State is estimated to be 7,300,000 from the National Population Census of 2006 projection [23].

### Subjects

Neonates with seizures, suspected seizures or with conditions associated with seizures were identified in the admission register. Their case files were retrieved from Health Information Department. Information extracted from the patients case file were age at admission, age at onset, gestational age of neonate, birth weight, mode of delivery, APGAR score, family history of seizures, social status of the parents, significant features on clinical examination and results of investigations done. Neonatal seizures were clinically defined according to Volpe’s classification [1] modified by Lombaso [22] as subtle, focal, multifocal clonic, focal tonic, generalized tonic and myoclonic seizures. The prevalence rate was taken as the number of neonates with seizures over the total number of neonates admitted during the period.

Case definition of some of the etiological factors:

Sepsis is a condition that happens when the body’s immune system has an extreme response to an infection, causing organ dysfunction and commonly manifesting with fever and other symptoms.

Bilirubin encephalopathy is bilirubin-induced neurological damage in a neonate with severe hyperbilirubinaemia Meningitis is an inflammation of the meninges, the membranes that surround the brain and the spinal cord manifesting with high fever, bulging anterior fontanelle and sometimes seizures.

Birth asphyxia is defined as the failure to establish breathing at birth, leading to organ dysfunction.

Discharged patients were follow-up for eighteen months.

### Data analysis

Data analysis was done using Statistical Package for the Social Sciences (SPSS 23). Categorical variables were summarized using frequencies, ratios and proportions. They were done within 95% confidence intervals. Continuous variables were summarized using means, standard deviation, median and inter-quartile range.

## Results

**Fig. 1:**
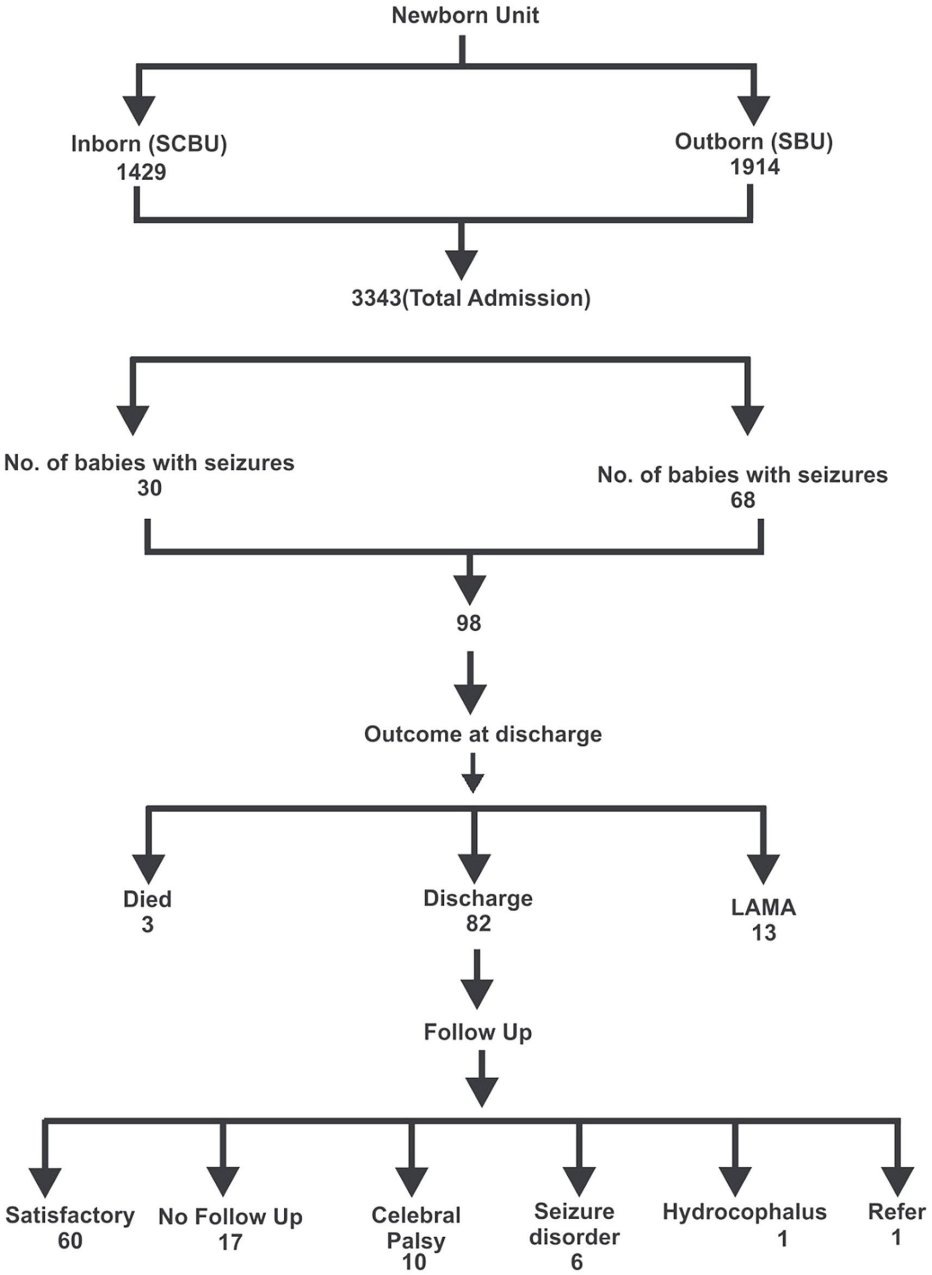
Population flowchart of newborn with seizures and its outcome

Figure 1 shows that ninety eight (98) of 3343 of newborns admitted from May 2013 to June 2018 had neonatal seizures, giving prevalence 2.93%. Out of the 7,583 neonates delivered in the hospital, 30 had seizures giving an incidence of 3.95%/1000 live birth. The median weight of neonates presenting with neonatal seizures was 3.0 kg (± 0.79SD).

In the preterm group, the commonest cause of neonatal seizure was hypoglycaemia while in term babies; birth asphyxia was the commonest cause of neonatal seizures. Subtle seizure/bizarre movements were still the commonest seizure pattern in both preterm (50%) and term babies (32.43%) respectively but they constituted a much high proportion in the preterm group.

In the follow up, there was almost equal proportion of satisfactory outcome of neonates whose period of onset of seizures were within the first three days of life (61.90%) and those whose period of onset were beyond three days of life (65.62). However even as there were varying degrees of complications amongst both groups, more complications were in the group whose period onset were within the first three days of life compared to those whose period of onset were beyond three days. Mortality also occurred in the group whose period of onset was within the first three days of life. Twelve (12) of the newborns with seizures had abnormal cerebrospinal fluid (CSF) findings. One of these died while one did not come for follow up. Of the remaining, 6(54.55) had satisfactory follow up and two (18.18%) developed hydrocephalus. One each developed seizure disorder, spastic hemiplegia and delayed developmental milestone.

Table Ia showed that 62% (62%) of the babies had neonatal seizures in the first three days of life while in about 28% (27.55%) of the babies, seizures occurred after age of seven days of life. 91% (91%) of the seizures occurred in term babies. There was slight predominance of males (57.1%) neonates who had seizures. Babies with low APGAR scores (55.1%) contributed more to seizures than babies who had normal APGAR score. 52% of neonates with seizures had feeding delays compared to about forty seven (47%) of babies with non-feeding delays.

Only about 26% (25.51) of the neonates had abnormal physical findings. Ninety-nine (98.9%) of the seizures were controlled within one week of admission. 79% (78.57%) of the neonates with seizures were discharged in good state while 13.27% of the babies were taken away by their parents against medical advice in the course of the management and their final outcome was not determined. Three (3.06%) were discharged with neurological deficit, three (3.06) died and one (1.02%) was discharged with hydrocephalous while one was referred. In the follow up, 63.16% had satisfactory follow up while 6.32% developed seizure disorders, 10.53% developed cerebral palsy. One (1.02%) was referred, while one developed hydrocephalus. There was no follow up for 17.89% of the neonates.

Table Ib shows that most of the mothers (89.80%) were booked and had Ante-natal care. Only 4% of the mothers of the neonates with seizures had hypertension while 90% of the mothers had no documented medical history. 48% of the mothers were nulliparous while 26.5% had delivered only once. More of the mothers (39%) were of middle social class while 29.6% were of higher social class. Seventy two (73.5%) of the babies were delivered via spontaneous vaginal delivery.

**Table I:** **a**: Neonatal variables of babies with seizures

Neonatal Indices	Frequency *n* = 98	Percentage (%)
**Age of onset of seizures**		
First two days	58	59.18
3rd day of life	3	3.06
4–7 days of life	10	10.21
More than 7 days	27	27.55
**Gestational Age(weeks)**		
Preterm (< 37)	8	8.16
Term (37–42)	89	90.82
Post term (> 42)	1	1.02
**Sex**		
Male	56	57.10
Female	42	42.90
**APGAR Score**		
< 3	30	39.80
4–6	15	15.30
≥ 7	44	44.90
**Feeding delay**		
Feeding delay	51	52.04
No feeding delay	47	47.16
**Physical examination findings**		
Increase tone	12	12.24
Bulging anterior fontanelle	13	13.27
No abnormal findings	73	74.49
**Duration of Seizures**		
Less than one week	97	98.98
Longer than one week	1	1.02
**Outcome at discharge**		
Discharged in good state	77	78.57
Died	3	3.06
Left Against Medical advice	13	13.27
Discharged with Neurological defect	3	3.06
Referred	1	1.02
Discharged with hydrocephalous	1	1.02
**Follow up after discharge**		
Satisfactory follow up	60	63.16
Seizure disorder	6	6.32
Cerebral palsy	10	10.53
No follow	17	17.89
Referred	1	1.05
Developed Hydrocephalous	1	1.05

**Table I:** **b.** Maternal variables of mothers of babies with seizures

Maternal indices	Frequency *n* = 98	Percentage (%)
**Booking status**		
Booked	88	89.80
Unbooked	10	10.20
**Medical History**		
Gestational diabetes	2	2.4
Hypertension	4	4.08
Retroviral disease	1	1.02
Post- partum Haemorrhage	1	1.02
Severe anaemia	1	1.02
Malaria in pregnancy	1	1.02
No medical history of illness	88	89.8
**Parity**		
Nulliparous	47	48
1	26	26.50
2	14	14.30
3	6	6.10
4	5	5.10
**Social Class**		
1	7	7.10
2	22	22.50
3	38	38.80
4	29	29.60
5	2	2.00
**Mode of delivery**		
Spontaneous	72	73.50
Caesarian section	24	24.50
Assisted vaginal delivery	2	2.00

Table II shows that risk factors for sepsis (64.18%) and low APGAR scores (26.81%) constituted the major risk factors for neonatal seizures. Other contributory risk factors were malpresentation (2.98%) and prematurity (2.24%).

**Table II:** Risk factors for neonatal seizures

Risk Factors	Frequency (*n*-134)	Percentage (%)
Low APGAR Score	36	26.86
Risk factors for sepsis	86	64.18
Intra-uterine growth retardation	1	0.75
Malpresentation	4	2.98
Postmaturity	1	0.75
Meconium Aspiration	1	0.75
Prematurity	3	2.24
Maternal Gestational Diabetes	1	0.75
Pre-eclampsia /eclampsia	1	0.75

### Table III, IV

Birth asphyxia constituted the singly commonest cause of neonatal seizures (44.90%). This was followed by infective causes [(sepsis 16.33% and meningitis 16.33%)]. In concert with other causes, sepsis constituted the highest cause of neonatal seizures. This was followed by birth asphyxia (24.75) and meningitis (16.83). Metabolic causes like hypoglycaemia and hypocalcaemia also contributed to neonatal seizures.

Most of the neonatal seizures (57.33%) occurred in the first two days of life. Birth asphyxia constituted the highest cause of neonatal seizures (48.84%) in the first two days of life while the infective causes-sepsis (43.90%) and meningitis (31.71%) were the most important causes of neonatal seizures after seven days of life.

**Table III:** Distribution of causes of neonatal seizures singly and in concert

Singly			In concert		
Causes	Frequency (*n* = 49)	Percent (%)	Causes	Frequency (*n* = 101)	Percent (%)
Hypoglycaemia	7	14.28	Sepsis	29	28.71
Sepsis	8	16.33	Birth Asphyxia	25	24.75
Birth Asphyxia	22	44.90	Meningitis	17	16.83
Meningitis	8	16.33	Bilirubin encephalopathy	2	1.98
Hypoglycaemia	3	6.12	Hypocalcaemia	7	6.93
Benign Sleep myoclonus	1	2.04	Hypoglycaemia	11	10.89
			Hypernatramia	2	1.98
			Hyponatramia	6	5.94
			Benign sleep Myoclonus	2	1.98

**Table IV:** Distribution of causes of seizures by age of onset

1st 2 Days			3rd Day			4–7 Days			> 7 days		
Causes	Frequency (*n* = 86)	Percentage	Causes	Frequency (*n* = 4)	Percentage	Causes	Frequency (*n* = 19)	Percentage	Causes	Frequency (*n* = 41)	Percentage
Birth Asphyxia	42	48.84	Birth Asphyxia	1	25	Birth Asphyxia	2	10.53	Sepsis	18	43.90
Hypoglycaemia	11	12.79	Hypoglycaemia	2	50	Meningitis	7	36.84	Birth Asphyxia	2	4.88
Meningitis	5	5.81	Benign Sleep Myoclonus	1	25	Bilirubin encephalopathy	1	5.26	Meningitis	13	31.71
Sepsis	15	17.44				Sepsis	4	21.05	Hypoglycaemia	3	7.32
Hypocalcaemia	7	8.14				Hypoglycaemia	2	10.53	Hypernatramia	1	2.44
Benign Sleep Myoclonus	2	2.33				Hypocalcaemia	2	10.53	Hyponatramia	1	2.44
Hyponatramia	3	3.49				Hyponatramia	1	5.26	Bilirubin encephalopathy	1	2.44
Hypernatramia	1	1.16							Hypocalcaemia	1	2.44

### Table V, VI

Bizarre movements and other subtle seizures were the most common seizure type (34.15%). This was followed by focal seizures (26.83%) being almost in the same proportion as the generalized pattern (24.39%). Generalized seizure type (25.71%) was the commonest seizure type occurring within the first two days of life. This was followed by focal (23.81%) and tonic seizure type (13.33%) respectively. Twitching of the corner of the mouth/lips (11.43%), blinking of the eyes (4.76%) and autonomic imbalance (3.81%) were also common during this period of onset. After age of onset of seven days, generalized (26.32%) and focal seizures type (26.32%) were the commonest seizure types. This was followed by tonic seizures type. Crying (5.26%) and grinning / facial twitching (5.26%) were also common subtle seizures type occurring after the age of onset of seven days.

**Table V:** Distribution of pattern/type of seizures

Clinical Pattern	Frequency (*n* = 164)	Percentage (%)
Focal Seizures	44	26.83
Tonic Posturing 3 with tonic deviation of neck	24	14.63
Generalized	40	24.39
7 with clonic component		
3 with tonic component		
Bizarre movement and other subtle seizures	56	34.15

**Table VI:** Distribution of seizure type by age of onset

Less than 2 days			Day 3			4–7 Days			More than 7days		
Seizure type	Frequency (*n* = 105)	Percentage	Seizure type	Frequency (*n* = 9)	Percentage	Seizure type	Frequency (*n* = 12)	Percentage	Seizure type	Frequency (*n* = 38)	Percentage
Tonic	14	13.33	Tonic	1	11.11	Tonic	2	16.67	Tonic	7	18.42
Generalized	27	25.71	Generalized	1	11.11	Focal	2	16.67	Generalized	10	26.32
Rolling of eyes	5	4.76	Rolling of eyes	3	33.33	Generalized	5	41.67	Rolling of eyes	4	10.53
Crying	7	6.67	Focal	4	44.44	Rolling of eyes	1	8.33	Focal	10	26.32
Focal	25	23.81				Crying	1	8.33	Blinking of eyes	1	2.63
Twitching of mouth/lips	12	11.43				Focal	1	8.33	Crying	2	5.26
Blinking of eyes	5	4.76							Sucking	1	2.63
Lip smacking	1	0.95							Grinning / facial Twitching	2	5.26
Chewing	2	1.90									
Bicycle	1	0.95									
Autonomic imbalance	4	3.81									
Grinning/ twitching of face	2	1.90									

## Discussion

The prevalence of neonatal seizures of 2.93% was comparable to that of Asindi [19] documented three decades ago in Calabar and also comparable to the revisit of the study eleven years later by Udo et al. [20] in the same hospital. The incidence of 3.95 was comparable to that of Omene et al. [18] in Benin who recorded an incidence of 3.15 per 1000 live birth. These studies were all in the South-South region of Nigeria and similar social, cultural factors may have been operative in the causation of seizures in the same zone. The prevalence was however in contrast to a much higher figure recorded by Kuti et al. [7] in south-west Nigeria. Majority of the newborns with seizures in this study were term neonates with only 8.2% of them being preterm. This was at variance with that of Omene et al. [19] and others [4, 12, 13, 14] who found preterm babies to be more significantly common among the babies who had neonatal seizures. The high prevalence of neonatal seizures among terms babies in our study may be because most of the patients were a cohort of hospitalized babies with high morbidities impacting on the brain and causing brain injuries thereby producing the seizures. Also, seizures in newborns being of paroxysmal behavior and caused by hyper-synchronous group of neurons do occur in pre-terms and may actually be underestimated in our environment where there is no facility such as video-electroencephalography (V-EEG) and pharmacological paralyzing of neonates for EEG study is not routinely done here. In keeping with the fact that most of the babies in this study were term babies, the median weight of the babies who had neonatal seizures was 3 kg. This was equally at variance with most studies where seizures were more reported amongst low birth weight (LBW) babies [8, 11, 14].

Neonatal seizures are rarely idiopathic therefore their causes must be sought for. There are many causes of neonatal seizures but most seizures result from only few disorders. There can be distinguishing characteristic clinical features such as age of onset, type of seizures, clinical status of the newborns and clues during the early management phase that can be used to identify these disorders. Birth asphyxia and infective causes either singly or in concert with other concomitant etiologic factors contributed more to the causation of neonatal seizures in the index study. Birth asphyxia was the most common single cause of neonatal seizures, while sepsis in concert with other etiologic factors were the most important combined causes of neonatal seizures. This was hardly surprising as risk factors for sepsis and low APGAR scores were the important risk factors for neonatal seizures. The etiologic factors in this study are not too different from other studies where post-hypoxic encephalopathy, metabolic causes and infections were documented [7, 19, 20].

Birth asphyxia had been a foremost cause of neonatal seizures in developed countries in the past [24, 25] but there has been a decline because of improved obstetric practices and improved neonatal care. Similarly birth asphyxia was a leading cause of neonatal seizures in the developing countries [4, 19, 20, 26] Developing countries, Nigeria inclusive, are still confronted with lack of proper referral system, [26] lack of manpower, basic facilities in hospitals, lack of proper neonatal resuscitation training and retraining [19] and deliveries are still being undertaken in prayer houses, churches and at home by the Traditional birth attendants [27]. These factors particularly home deliveries [28] are operative in Akwa Ibom. Infections and birth asphyxia are the consequences where these factors are operative [19, 29] as they were in our study site. The magnitude of asphyxia as a leading cause of neonatal seizures in the index study compared to others earlier stated was less, probably because there has been some improvement in health care delivery over the years. Other concomitant operative causative factors of neonatal seizures such as hypoglycemia and hypocalcaemia may be due to catabolic stress occurring in situations like sepsis, meningitis or hypoxic-ischemic encephalopathy.

Birth asphyxia, the singly most common cause of neonatal seizures in our study was responsible for the neonatal seizures occurring within the first two days of life. Hypoglycemia tended to follow the same time course although a few still occurred in ill babies who had seizures whose period of onset was beyond four days. Hypocalcaemia tended show a bimodal chronologic distribution, the early ones causing seizures within the first two days of life and the late ones, causing seizures at the age of one week of life or beyond. Seizures due to infections were present at any time, but more commonly, in those with intra-cranial infectious, the seizures began during the later part of first week, usually in the context of systemic sepsis. Age of onset in relation to etiology in this study is an agreement with other studies [19, 24]. Seizures occurring as a result of asphyxia were found in other studies to occur in the first three days of life [19, 25].

Bizarre movements and other subtle seizures constituted the most common seizure type in the general population in the index study. This was followed by focal seizures and generalized seizures in almost the same proportion. Subtle / bizarre patterns were also the commonest seizures pattern in both the preterm group and in the term neonates. The seizures were considered subtle because their clinical manifestations were usually overlooked. They were usually stereotypical, repetitive and were difficult to distinguish from normal behaviors of the inter-ictal moments or physiological phenomena of the newborn. The common subtle seizures were twitching of the corner of mouth/lips-smacking, grinning/twitching of face, chewing and crying. We did not have the benefit of EEG to either consistently monitor or singly analyze the subtle seizures to establish their true ictal activities even though simultaneous monitoring of subtle seizures has not shown consistent electrographic discharges and at times those movements have been shown to be “brain stem release phenomena [17].

The mortality of 3.06% in the index study was considerably lower than those of Omene, [18] Asindi, [19] Udo, [20] in the same geo-political region and also lower than those of Kuti, [7] Owa, [29] Ogunlesi, [30] Adebami, [31] in the South-West region of Nigeria. It also contrasted significantly with those of studies from other parts of the world [10, 32]. This might be because a significant proportion of these neonates, especially some, who were refractory to anti-convulsants were taken away against medical advice and their final outcome were not determined. There has also been a significant improvement in managing these cases over the years.

Among the survivors who were discharged and were subsequently followed up (for 18 months), 17.89% of them had adverse outcome such as seizure disorder, cerebral palsies with spastic hemiplegia, delayed milestone, and hydrocephalus. Lack of follow up in some especially those who left against medical advice, made it difficult to know the actual proportion of those with adverse outcome. The percentage of adverse outcome including mental retardation may actually have been higher if the patients were followed up for a much longer time.

In the follow up, there was an almost equal proportion of satisfactory outcome amongst newborns whose period of onset were within the first three days of life (61.90%) and those whose period of onset were beyond three days of life (65.63%). More Serious complications were however observed in the first group (period of onset 3 within the first three days of life), thereby suggesting that early onset seizures were associated with poor prognosis. The mortalities recorded were also in this group. The sequelae observed in this group might relate to the acuteness/criticalness of seizures during this period, where resultant hypoventilation and poor perfusion might have compromised the neonate who, if inadequately or not properly managed could lead to cardiovascular collapse, decreased cerebral blood flow, with increase risk of hypoxic-ischaemic injury.

Accompanying hypercabia could possibly lead to rise in cerebral blood flow and an increased risk of intra-cranial haemorrhage. In the acute phase, blood pressure might also be raised and this might increase cerebral blood flow, leading to intra-cranial haemorrhage. All these would lead to brain damage and possibly death. More than half of the neonates with seizures who had abnormal cerebrospinal fluid were discharged without neurological deficit while some were discharged with neurological deficits and a few were taken away against medical advice. During the follow up of the babies who were discharged, more than half had satisfactory follow up, while some developed hydrocephalus and the remaining ones each developed seizure disorder and delayed developmental milestone.

## Conclusion

The prevalence of neonatal seizures in Uyo was 2.93%. Birth asphyxia and infective causes either singly or in concert with other etiological causes are responsible for neonatal seizures. More complications were observed in babies where period of onset were within the first days of life.

**The limitations of the study were**:

1. It was not population-based study. Hospital-based studies tend to report higher incidence of neonatal seizures because most of the affected newborns were prone to central nervous system injuries and these predisposed them to seizures.
2. Improved quality of diagnosis of neonatal seizures would have strengthened the study. Confirmation of neonatal seizures depends on the use of EEG especially on those infants who are paralyzed pharmacologically, even though there are issues in interpreting inter-ictal EEG in this age group and its diagnostic value is often limited. EEG was not done here and there was limited use of neuro-imaging on account of cost. Clinical diagnosis alone is unreliable, thereby leading to misdiagnosis and therefore to inappropriate use of anticonvulsants and this also leads to inability to accurately predict outcome. The risk of overestimation of neonatal seizures was an important limitation since it was based on clinical approach and a few biochemical considerations.
3. Neonates at risk of seizures but did not have seizures were not investigated.
4. The sample size was small.
5. The follow up period was short and so did not offer opportunities for the evaluation of long term morbidities such as cerebral palsy, intellectual disability and epilepsy.

## Electronic supplementary material

Below is the link to the electronic supplementary material.

Supplementary Material 1

## Data Availability

The datasets used and/or analysed during the current study are available from the corresponding author on reasonable request.

## Abbreviations

CNS: central nervous system
CSF: Cerebrospinal fluid
EEG: Electro-encephalography
LBW: low birth weight
SBU: Sick Baby Unit
SCBU: Special Care Baby Unit
SD: Standard Deviation
SPSS: Statistical Package for the Social Sciences
UUTH: University of Uyo Teaching Hospital
V: EEG-video-electroencephalography
