# Supplementary material for: Neonatal seizures in Uyo: the burden, etiological factors and outcome
Source: BMC Pediatr. 2025 Jun 6;25:460. doi: 10.1186/s12887-025-05742-1 (PMC12142816; doi:10.1186/s12887-025-05742-1)
Supplement: Supplementary file 1 — Supplementary Material 1 [file 12887_2025_5742_MOESM1_ESM.docx]

**Social classification using the scheme proposed by Oyedeji**

| **Class** | **Occupation** | **Education level** |
| --- | --- | --- |
| 1 | Senior public servants, professionals, managers, large scale traders, businessmen and contractors | University graduates or equivalents |
| 2 | Intermediate grade public servants and senior schools teachers | School certificate (ordinary level GCE) holders who have teaching or other professional training. |
| 3 | Junior school teachers, drivers, artisans | School certificate holders, or grade II teachers certificate holders or equivalents. |
| 4 | Petty traders, labourer, messengers and similar grades | Modern 3 and primary school certificate. |
| 5 | Unemployment, students, full time, housewife and subsistence farmers | Those who either just read and write or are illiterate |
